# The Diagnosis and Treatment of Whooping Cough

**Published:** 1881-10

**Authors:** Robert Lee


### The Diagnosis and Treatment of Whooping Cough.

By Robert Lee, M. D.

The history of whooping cough, or chin cough, would almost lead to the belief that the disease must have existed long before it was recognized as a specific malady and designated by a special name.

I do not propose to enter fully into the historical narrative, but there are two or three facts which I shall briefly state, the explanation of which is worthy of consideration.

In 1678, or about two hundred years ago, there was a mortality in London of 20,471, amongst which one case of whooping cough and two cases of chin cough are mentioned, this being the first entry in the returns. For fifty years after chin cough and whooping cough were registered separately, as though they were different in their nature, till in 1730 they were entered together, when the mortality was 152 in 27,000, or 1 death in 177. Gradually the mortality increased, till it reached an average of 1 in 29 for London; and during the last few years the whole country has suffered, and now there is a higher mortality in the total of deaths in Great Britain than has ever been registered before. Are we to explain these facts by supposing that the disease has actually increased, or that we have been slow to recognize it? I shall say no more in detail on this point, except that it is a far more important one than might be imagined, and has a distinctly practical bearing on the questions of diagnosis and treatment.

It is now about nine years ago since I began to observe, with the opportunities afforded by the practice of a large children's hospital, the various features of whooping cough, noting any facts which appeared to be important, as they occurred from time to time. It is probable that those who have been occupied in similar practice will agree with me that among the poorer classes of a large city the average of whooping cough cases may be estimated at nearly 10 per cent. of all cases under treatment.

The first point which forces itself on the attention is that whooping cough is far more prevalent in infancy than is generally supposed. Sir Thomas Watson's opinion that the disease is rare in very early life is certainly not confirmed by my experience; and as this opinion has been evidently received and restated in works on medicine—as, for example, in one of the most recent and popular in America, which says that the disease rarely affects children before weaning—it is well to direct attention to a possible source of error in diagnosis. I am quite satisfied that if an infant of only a few days old be in the same apartment with another child suffering from whooping cough, whether it be weaned or not, it will in all probability take the disease; at least, its age will not protect it. And just as the mortality is greater among children from whooping cough before the age of twelve months than later, so the occurrence of the disease is more general.

The next point of interest is the degree to which whooping cough is infectious. Of late the possibility has been discussed of the principle of imitation being involved in the symptoms, and there has been a tendency to refer them to nervous derangements, much in the same way that the disease found its place in the nosology of Cullen, along with asthma, in the class of spasmodic diseases. But when it is clearly shown that whooping cough is very infectious, and that it must be regarded properly to belong to the class of which scarlatina and measles are typical members, I need hardly remark on the little practical use of discussing how far a child may control the cough by effort, or what is the precise physiological explanation of the nervous symptoms. It will be time for that when we have ascertained how to prevent the spread of the malady and have diminished its mortality.

The following are a few cases out of a large number of which I made notes at the time that the subject of the infectious character of the disease was under investigation, and here I may remark that every precaution is taken to separate the whooping-cough cases from others.

In 1876 a child aged eighteen months was brought, on May 11th, to be treated for convulsions, being otherwise well. On July 3, whooping cough was clearly diagnosed—that is, fifty-three days later.

On May 22, a child was brought with enlarged cervical glands. On June 12, the first symptoms of whooping cough showed themselves. On July 10th a brother of the child, not attending the hospital, had the disease.

A child (male, aged ten weeks) was brought to the hospital with eczema of the head and body on May 29th. Had whooping cough on July 10th. Another member of the same family was brought with it. A boy, aged thirty-seven months, was brought in for congestion of the brain on June 15th. On July 20th the disease was well marked. A child (male, and seven weeks), emaciated from ill-feeding, was brought in on May 11th. On June 19th the disease first showed itself. The mother would not allow that it had whooping cough till four weeks after this. Another child was brought for rickets on May 22nd, and on July 14th the disease was well marked.

In my note-book for 1878 there are the following instances: A child (female, aged four weeks) was brought with congenital syphilis on May 30th; the disease was well marked on June 20th. Another on the same day with eczema, and after the same period showed symptoms of the disease. Another child (nine months old) was brought with eczema on Oct. 19th, and on Nov. 24th there was well-marked whooping cough. Another child (aged three weeks) was brought with obstinate constipation on Nov. 3, and on Nov. 20th symptoms of whooping cough first showed themselves. There are eleven more cases, of the respective ages of eighteen months, fourteen months, three months, six weeks, three months, eight months, seven months, four months, thirteen months, thirteen months, and eighteen months, none showing any symptoms of the disease when they were first brought, and suffering from rickets, eczema, improper food, or other causes of illness.

Taking an average from these twenty-one cases, we have thirty-two days as the period which may elapse between exposure to infection and well-marked symptoms of the malady. Very frequently I have noted in cases the first symptoms of whooping cough from a week to a fortnight before the mother would admit the fact, and in stating the average at thirty-two days, I should deduct ten days or more for the obscure symptoms of the early stage of the malady, thus making the period of incubation generally three weeks.

It is singular to notice how obstinate most women are in denying that their children have whooping cough, so that their evidence or opinion is of little value. Among these cases there was one of a mother who caught the disease. She lost her infant, and had two other children ill with it. In another family two others at home caught it, one an infant five weeks old. In another the infant caught it, aged seven months.

Without dwelling long on the law of infection as regards adults, to judge from seven cases in hospital practice, and an equal number in private life, in which the father, mother, or nurse caught whooping cough a second time, I think we may conclude that close and constant exposure to the influence of infection is the almost invariable cause of such an occurrence.

The fact of all others which has appeared to me most singular, and certainly the most important in diagnosis, is the frequency with which whooping cough in very young children is mistaken for some other disorder, and this is entirely due to the belief that the symptom of the whoop must be present in order to satisfy the demands of diagnosis. Without the least hesitation, and without the least fear of exaggeration, I venture to express the opinion that infants whoop but rarely. Instead of expecting such a symptom, I am surprised when it occurs in a very young child, and, on reflection, am still more surprised that we should expect it to do so.

Many medical practitioners must have remarked this fact after some experience of family practice, though I could mention instances of personal friends who have been astonished that they had not noticed it till their own children were the subjects of their observation. That it was well known, and carefully noted years ago, is quite evident, since it was stated and taught by Dr. Cullen. "I have had instances," he says, "of a disease which, though evidently arising from the chin-cough contagion, never put on any other form than that of a common catarrh." And again, "when the disease beginning in the form of a catarrh is attended with fever and difficult breathing, and with little expectoration, it often proves fatal, without taking on the form of the whooping cough."

Now this is the form which the disease does assume in very young children, and which quite accounts for its being so dangerous in early life. It is not quite correct to say that it begins in the form of a catarrh. The disturbance of the system is more serious, and, if the question is asked of the parent whether the child has been exposed to cold, it is generally answered in the negative. The symptoms are those which more or less characterize the contagious maladies in their early though rather indefinite stage. The most marked certainly are refusal of food, prostration, restlessness, and loss of flesh, with more or less increase of temperature and quickness of respiration. We are inclined at one time to suspect pneumonia or capillary bronchitis, at another that the cause of the symptoms is dentition, or, if they are not severe, that they are due to some error in feeding. Sir Thomas Watson has followed Cullen when he says that "it begins with the symptoms of an ordinary catarrh arising from cold;" and, to show to what extent he considered the cough diagnostic of the malady, he says, "I should be slow to consider any case a genuine case of pertussis unless the characteristic paroxysms of whooping cough and the stridulous respiration were present."

There is a symptom which is a serious and frequent one in infants, and that is the occurrence of active diarrhœa. It is true that the diarrhœa is often preceded by vomiting, but, as a rule, when the diarrhœa begins the other symptoms abate, and one of the two events occurs: either the infant succumbs, in spite of all treatment, or the disease appears to exhaust itself and the symptoms do not return. The active treatment with purgatives, recommended by Sydenham, appears thus to be indicated to some extent by the natural course which the disease takes in such cases.

Sir Henry Holland pointed out the probable relation between whooping cough and what used to be called infantile remittent fever, and distinctly expressed the opinion that they would be found to depend on the same active cause.

Before passing on to the subject of treatment, there are one or two points which are deserving of notice. One, the most important, that in a family of two or three children, when circumstances compel them to be kept in the same room, and the conditions of ventilation are deficient, the duration and severity of the symptoms appear to increase, and the nurse or parent is liable to suffer, from which it would seem that the atmosphere of a close apartment may become more or less highly charged or saturated with the fomites of contagion. Another point is the usual increase of fever, difficulty of breathing, and cough during the night, and it is reasonable to inquire whether this may be due to somewhat similar causes to those which mark the stages of an attack of ague or to some difference in the conditions of the nervous system during the usual hours of sleep.

One point seems worthy of mention, and that is the probable explanation of the fact that sometimes all the symptoms of whooping cough will suddenly subside, and, after an interval of several months, reappear without any very definite reason, such as exposure to a second infection.

The subject of the treatment of this malady appears to me to be pre-eminently deserving of most serious attention. The infant mortality it has lately caused has grown to surprising importance, as the Registrar-General's Returns have made most evident. The remedies which have from time to time been recommended have been so numerous, that it is probable most practitioners would agree with Sydenham that, with some one exception, which in his case was active purging, they "have tried remedies of almost every other kind, and tried them in vain."

Failing any specific remedy, we are naturally compelled to treat symptoms, making use of such agents as are generally recognized as proper for the different morbid conditions of the patient. The sensible practitioner would regard any one remedy with considerable doubt, and, while admitting the value of some few in relieving the frequency and violence of the cough, would combine with them either a febrifuge, a purgative, or a tonic, as the state of the case demanded. Thus in the early stages we might prescribe, as is often done, a combination of belladonna, bromide of potassium, and squills or ipecacuanha; later, hydrocyanic acid or alum, or external applications, such as camphor, turpentine, or Roche's embrocation; and finally, when the cough seemed to resist all treatment, we should order with confidence a change of air. I recollect, some years ago, one evening a German merchant called upon me to inquire what remedy we used at the Children's Hospital, as some poor neighbors of his had told him of the good effects obtained by themselves and several others from the treatment. I informed him that we had no special remedies; but as he had come from some distance, and seemed much disappointed, I ventured to inquire what was the nature of the medicine prescribed. He said it was a mixture of cod-liver oil and steel. Now, there is only one mixture of this kind contained in our Pharmacopœia, so I had no difficulty in satisfying his wishes. But it certainly surprised me to hear that this combination, by no means an agreeable one, had obtained a reputation in whooping cough cases quite unknown to us. Since then I have used it largely, and with as much benefit as some far more esteemed preparations.

There has been for some years a popular notion that a specific remedy does exist, though in a form which is difficult of application. I refer to the influence stated to be exerted by the exhalations from the waste chambers which form part of a gas factory. From abroad we have statements which show that it is usual for children to be taken to these places at convenient times, and that "this is encouraged" by medical advice. I was informed by one of the physicians resident in Amsterdam that special arrangements were made for children suffering from whooping cough; and in our own city, at some gas-works in Holloway, for example, on certain days when the waste chambers are cleared, it is usual for many children to attend at the works with apparent benefit. The question which naturally suggests itself is this: What particular product of coal distillation would probably exert an influence on the contagion of whooping cough? I am not in a position to answer this question at present, nor do I see how to begin such an inquiry without the assistance of a scientific chemist who has made a special study of the subject. This I hope to obtain shortly. Applying, however, the general principles of the antiseptic treatment, and assuming that there are very likely several agents present in the waste products of coal distillation which would be more or less potent in this respect, I have used largely the common one, carbolic acid, combined with turpentine, benzoin, or one of the fragrant oils. This plan of treatment has become very general, and I have heard some go so far as to say that carbolic acid will cure whooping cough. This statement has come usually from those of limited experience, though sufficiently good results have been obtained to justify us in pursuing the line of reasoning which first suggested a trial of the remedy. Not long since I was assured by a distinguished member of our profession, who suffered severely from whooping cough, that the only remedy which afforded him relief was carbolic acid inhalation; that the favorite treatment with bromide of potassium and belladonna was tried with the result that for a few hours the cough was relieved after taking the first few doses, but in a short time the effect passed off, and was followed by increased violence of the cough and painful dryness of the throat. In adults the spasmodic cough is the most serious symptom of the disease, but in infants we have to fear the rapid wasting and frequent diarrhœa perhaps more than the cough. So that I am compelled to admit that the use of carbolic acid inhalation must be limited, but within those limits it may be said to have a special effect. I have no doubt that some have tried the administration of the acid internally in diluted solutions, but in my own experience the results have not been such as to warrant any definite opinion. I have also tried sulphur in frequent and small doses, and have had fairly good results. There is no doubt that, as a general rule, the treatment recommended by Sydenham of purging actively is a wise one, for the distress in respiration and cough is certainly diminished when the mucous discharges from the bowels are encouraged. It may also be observed that a spontaneous diarrhœa in whooping cough is not to be controlled by opiates or astringents. It appears to me, then, that we have two distinct symptoms to deal with, and that to a certain extent they are under control. With regard to the laryngeal spasm, there is no doubt we can give relief by the inhalation of carbolic acid; with regard to the diarrhœa, I think the best results are obtained, not by attempting to check the discharges by opiates or astringents, but by the administration of quinine and iron in full doses, and by the application of such combinations as Roche's embrocation, a simple compound of the oils of turpentine and amber, to the surface of the abdomen. There is no doubt that infants at the breast are in less danger than those brought up by hand.

In conclusion, I would suggest a trial of other volatile agents for the purpose of inhalation, such as turpentine, creosote, oil of amber, benzoin, etc. The common plan of stirring some tar with a hot poker is certainly attended with benefit, and is largely used in my hospital practice. It is only necessary to observe that if we are to expect relief from this kind of treatment, it should be carried out on proper principles. It is of no practical use to order twenty or thirty drops of carbolic acid to be added to a pint of hot water, and the steam to be inhaled, for the quantity of acid which evaporates is only proportionate to the amount of water given off as steam, and under such conditions it is evident the amount would be infinitesimally small. —*London Lancet.*

